# Prevalence of metabolic syndrome and diabetes mellitus type-2 and their association with intake of dairy and legume in Andean communities of Ecuador

**DOI:** 10.1371/journal.pone.0254812

**Published:** 2021-07-23

**Authors:** Manuel E. Baldeón, Camilo Felix, Marco Fornasini, Federico Zertuche, Carolina Largo, María José Paucar, Liz Ponce, Sumathy Rangarajan, Salim Yusuf, Patricio López-Jaramillo

**Affiliations:** 1 Facultad de Ciencias de la Salud Eugenio Espejo, Universidad UTE, Quito, Ecuador; 2 Population Health Research Institute, McMaster University and Hamilton Health Sciences, Hamilton, Canada; 3 Masira Research Institute, Medical School, Santander University (UDES), Santander, Colombia; Instituto Nacional de Geriatria, MEXICO

## Abstract

Metabolic syndrome (MetS) and type 2 diabetes (T2D) are metabolic alterations associated with high morbidity and mortality, particularly in low and middle-income countries. Diet has a significant impact on the risk to develop MetS and T2D; in this regard, consumption of fruits, vegetables, and protein rich foods (from plant and animals) are important to prevent and manage these pathologies. There are limited studies regarding the potential association between Andean foods rich in proteins and the presence of cardio-metabolic conditions in Ecuador. It is necessary to develop new low-cost, local-culturally acceptable strategies to reduce the burden of cardio-metabolic diseases. We describe the prevalence (baseline data) of MetS and T2D in the Ecuadorian cohort of the Prospective Urban and Rural Epidemiology (PURE) study and their potential association with the consumption of protein rich foods, including beef, white meat, dairy and legumes. In this cross-sectional study, we assessed 1,997 individuals aged 35–70 years (mean age 51 years, 72% women), included in the Ecuadorian cohort of the PURE study, from February to December 2018. The prevalence of MetS was 42% for male and 44% for female participants; the prevalence of T2D was 9% for male and 10% for female. Metabolic syndrome and T2D were more common in women older than 50 years of age with primary education or less, low economic income, and with obesity; MetS was more frequent in the rural area while T2D was more frequent in the urban area. Using logistic regression analysis, we observed a significant protective effect of higher consumption of dairy and legumes in the prevalence of MetS and T2D compared with low consumption. It will be important to develop policies for ample production and consumption of protein rich foods such as legumes and dairy, part of traditional diets, to reduce the burden of cardio-metabolic diseases.

## Introduction

Cardiometabolic diseases including metabolic syndrome (MetS), obesity, and diabetes mellitus type-2 (T2D) are an increasing health problems worldwide [1, 2]. In 2019, approximately 463 million people around the world lived with T2D, primarily in low and middle-income countries [2]. In Latin America, the prevalence of T2D in adult population ranges from 8% to 10% [3]. It has been estimated that the total cost for diabetes care in Latin America ranged from US$ 102 to 123 billion; and that per capita cost was between US$ 1088 and US$ 1818 by 2015 [4]. According to The Ecuadorian National Health and Nutrition Survey (ENSANUT), the prevalence of T2D was 10% in individuals between 30 to 59 years in 2012 [5]. Metabolic abnormalities associated with MetS include obesity; high fasting plasma glucose and triglycerides; low high density lipoprotein (HDL)-cholesterol; and high blood pressure [6]. Subjects with MetS have increased risk to develop T2D and cardiovascular diseases that are main causes of morbidity and mortality [6]. The Ecuadorian National Institute of Statistics and Censuses (INEC) reported that T2D was the second most frequent cause of death in 2018 accounting for 6.6% of all registered deaths [7]. These data indicate that metabolic diseases are a common public health problem that primarily affect low and middle-income countries that further diminish their opportunities for development.

Several risk factors have been associated with the development of obesity, MetS, and T2D including a sedentary lifestyle, unhealthy diets, and poor socioeconomic status [8]. According to the American Diabetes Association, diet has a significant impact on the risk of T2D; in this sense, consumption of whole grains, legumes, nuts, fruits and vegetables are important to prevent and manage T2D [9]. The prospective urban rural epidemiology (PURE) study has shown in 18 low-middle income countries, not including Ecuador, that high consumption of legumes (3 to 4 servings/day) is associated with a 22% decrease in total- and non-cardiovascular mortality risks [10]. Our group has also shown that consumption of *Lupinus mutabillis*, an Andean legume commonly consumed in Ecuador, improves metabolic control in individuals with T2D, reducing serum concentrations of glycated hemoglobin and decreasing blood pressure [11]. In vitro studies show that *L*. *mutabilis* hypoglycemic effects could be attributed to the enzymatic inhibition of dipeptidyl peptidase-IV, inhibition of hepatic gluconeogenesis, and decreased insulin resistance [12]. Also, previous data from the international cohort of the PURE study has shown that consumption of more than 2 servings of total dairy per day is associated with a lower risk of mortality compared with no dairy intake [13]; and a lower frequency of metabolic syndrome, T2D and hypertension [14]. These studies support the recommendation to promote the consumption of protein rich foods for the general population. Nevertheless, there are limited studies regarding the potential association between local foods rich in proteins, including legumes, and the presence of cardio-metabolic conditions in Ecuador. It is necessary to develop new and culturally acceptable strategies to increase protein consumption by the general population in order to decrease the burden of cardio-metabolic diseases worldwide. It is also necessary to strength scientific evidence regarding the cardiometabolic benefits of protein consumption. This evidence will support public policies to promote the local production and consumption of environment friendly and culturally acceptable foods such as legumes (*L*. *mutabilis*) to prevent and to treat cardio-metabolic conditions. The objective of the present study was to describe the prevalence of cardiometabolic conditions including MetS and T2D in the Ecuadorian cohort of the PURE study and their potential association with the consumption of protein rich foods, including beef, white meat, dairy and legumes.

## Subjects and method*s*

### Study design and population

This cross-sectional study analyzed baseline data of the Ecuadorian cohort (n = 2020) of the international PURE epidemiological study coordinated by the Population Health Research Institute (Hamilton, Ontario, Canada) and 1997 participants with complete data were included in the final analysis. The design and methodology of the PURE study has been published previously [15]. Volunteers were recruited from four rural (n = 825) and five urban (n = 1172) communities in the province of Pichincha, Ecuador, located in the Andean mountains with a mean altitude of 2,800 meters over sea level from February to December 2018. A multi-stage convenience sample was used to recruit the study population. The Pichincha-province was selected due to the convenience of logistics and facilities, and because it is the second largest province of the country. To select urban communities, we used a list from the Metropolitan District of Quito, the capital city of Ecuador [16], and to select rural communities we used the criteria previously defined in the PURE study [15]. Each selected community was defined as a geographical area inhabited with individuals with similar ethnic and cultural characteristics. In order to efficiently recruit participants within each community, we approached suitable public and private institutions willing to participate in the study. Communities were selected based on the number of total inhabitants (maximum 5,000 in rural areas) and the capability to recruit at least 150 volunteers between 35 and 70 years of age. In order to select the household unit, we used 2 strategies: 1. subjects from households were personally invited to participate based on local maps close to the recruitment sites; and 2. word of mouth invitations.

### Ethics

The Ethics Committee of Universidad San Francisco de Quito approved the study (Code number: 2017-074E). Selected volunteers that signed a written informed consent were included in the study.

### Sociodemographic variables and clinical history

Sociodemographic characteristics and medical history were collected according to the PURE protocol using standardized questionnaires [15]. The variable of education was categorized by years of schooling as none/primary (0–6 years), high school/secondary (> 6–12 years), and university/technical school (>12 years). Household income was divided in tertiles as low (<387 USD/month), medium (387–699 USD/month) and high (≥700 USD/month).

### Assessment of nutrients intake

Habitual food intake was recorded using validated food frequency questionnaires (FFQ) that have been published previously [10, 13]. Consumption of legumes (beans, lentils, peas, fava beans, chickpeas, and lupins) was recorded by the amount of intake each time (once, twice, or more than three-times per day; once, 2 to 4, or 5 to 6 times per week; and one to three times per month) and never or almost never. To convert food into nutrients, we used country-specific nutrient databases for Ecuador (ENSANUT) with information about macronutrients and micronutrients that were mainly based on the United States Department of Agriculture (USDA) food-composition database [5]. This data base was also used to estimate total caloric intake.

### Blood biochemistry analysis and metabolic syndrome and diabetes diagnosis

Diabetes was defined with a fasting blood glucose level of ≥ 126 mg/dL, or when participants reported taking glucose-reducing drugs [17]. Metabolic syndrome was diagnosed when participants had at least three of the following conditions: waist circumference greater than 94 cm for men, greater than 80 cm for women; fasting triglycerides ≥ 150 mg/dL, HDL-cholesterol < 40 mg/dL (male), < 50 mg/dL (female); systolic blood pressure (SBP) ≥130, or diastolic blood pressure (DBP) ≥85 mm Hg; and fasting glucose >100 mg/dL [17].

Glucose and lipid profile (total cholesterol, LDL-cholesterol, HDL-cholesterol, and triglycerides) were measured on a HUMANSTAR 80, (Wiesbaden, Germany) using standard reagents (Human Diagnostics Worldwide, Wiesbaden, Germany). Blood biochemistry analyses were carried out after 8 to12 hours of fasting; and quality controls were used for every assay.

### Blood pressure measurement

Hypertension was defined with at least one of the following conditions: use of antihypertensive medication or a measured blood pressure (BP) with mean of SBP ≥ 140 mmHg and/or a mean of DBP ≥ 90 mmHg. Blood pressure was measured using a calibrated automatic digital device (Omron HEM-RML31; Omron Healthcare Co. Ltd., Scarborough, Ontario, Canada) by trained personnel. Previous to the measurement, participants sat down for at least 5 minutes; patients were instructed not to eat, smoke cigarettes, or do physical activity (i.e. claim stairs), 30 minutes prior to BP measurements. To measure BP, volunteers sat in upright position and had their right arm supported at heart level; their brachial artery was located and both SBP and DBP were taken twice with a time period of 3 minutes between each measurement; appropriate cuff sizes were used based on individual´s phenotype. The average of measurements was used for analysis [17].

### Anthropometric measurements

Weight was measured by trained personal with a calibrated scale (Body Composition Monitor, Tanita BC554, Tanita Corporation, Maeno-cho, Itabashi-ku, Tokio, Japan) according to the PURE protocol [15].

Overweight was diagnosed with a BMI ≥ 25 and <30, and obesity with a BMI ≥ 30. Abdominal obesity was determined with a waist/hip ratio (WHR) in men ≥ 0.90 and in women ≥ 0.85. For height measurements, a stadiometer was mounted vertically above a hard, flat surface, following vendor instructions (H.A. Kidd & Company, 3021060B, China). Height was measured to the nearest 0.1 cm. Waist and hip circumferences were measured using a not expandable tape (VWR International, WLS3678-DD, China). During anthropometric measurements, volunteers had minimal clothing and no shoes.

### Statistical analyses

For the statistical analysis 1997 volunteers with complete data were included. With this sample size we were able to calculate the prevalence of the cardio-metabolic diseases studied assuming a prevalence of 50%, an error level less than 2.5%, and a confidence level greater than 95%. Prevalence values were estimated with their 95% confidence intervals using a binomial score-test. Chi-Squared tests were used to compare differences in categorial variables between groups with or without MetS, T2D, and overweight/obesity [14]. Means and standard deviations were used to summarize the distribution of clinical parameters for participants with and without MetS and T2D as well as the total population. We used t-test p-values to compare differences in continuous variables between groups. To analyze protein intake and its association with cardio-metabolic diseases we used the following intake categories: low, ≤ 0.5; moderate 0.51 to 1, and high >1 serving per day; standard serving sizes (such as a glass of milk, a cup of yoghurt, or a slice of cheese) were assigned for dairy and 150 g for cooked legumes [10, 13]. We used linear models to assess the relationship between laboratory measurements (HDL, LDL, triglycerides, glucose), clinical characteristics (SBP, DBP, WHR, BMI) and legume intake by computing adjusted means. Thus, we first estimated the coefficients of a linear model controlling for sex; age; BMI; waist-to-hip ratio; residency type; education; income levels; smoking and drinking habits; diet energy; physical activity; beef, dairy, white meat and legume consumption. Subsequently, we computed the estimated mean values for the laboratory and clinical variables shown in Table 3 for each level of legume intake [14]. To estimate the effect of legume intake ANOVA-like tests were used. Odds ratios (OR) with their 95% confidence intervals were computed using logistic regression models for the probability to have MetS, T2D, and hypertension. In all the models the predictors were sex, age, BMI, WHR, urban/rural residence, education, income, smoking and drinking habits, physical activity, energy from diet (including carbonated beverages), and protein intake (beef, dairy, white meat, and legume) consumption. All the functions used in the analyses were implemented in the statistical software R version 3.6. A p-value < 0.05 adjusted for multiple comparisons with the method of Benjamini Hochberg, was considered statistically significant.

## Results

### Characteristics of study population and prevalence of metabolic syndrome and type-2 diabetes

The characteristics of the study population are shown in Table 1. The majority of participants were women (72.1%); older than 50 years of age (51.3%); without university education (82.8%); with low or medium income (62.8%); the mean age of the study population was 51±10 years.

**Table 1:** Sociodemographic and anthropometric variables for individuals with metabolic syndrome, type-2 diabetes, and excess weight.

Variables	n	Metabolic Syndrome Prevalence (%)	[95% CI]	p-value	Diabetes mellitus Type-2 Prevalence (%)	[95% CI]	p-value	Overweight /Obesity Prevalence (%)	[95% CI]	p-value
**Sex**										
Male	558	42%	[38%, 46%]	Reference	9%	[7%, 11%]	Reference	74%	[70%, 77%]	Reference
Female	1439	44%	[42%, 47%]	0.403	10%	[9%, 12%]	0.440	79%	[77%, 81%]	0.033
**Age Group**										
≤ 50	972	33%	[29%, 35%]	Reference	5%	[3%, 6%]	Reference	75%	[73%, 78%]	Reference
> 50	1025	54%	[51%, 57%]	< 0.001	15%	[13%, 17%]	< 0.001	80%	[78%, 83%]	0.033
**Education Level**										
None, Primary	989	49%	[45%, 52%]	Reference	11%	[9%, 13%]	Reference	71%	[66%, 75%]	Reference
High School/Secondary	664	42%	[39%, 46%]	0.027	9%	[7%, 11%]	0.289	79%	[77%, 82%]	1
University / Trade School	344	33%	[28%, 38%]	< 0.001	8%	[5%, 11%]	0.211	79%	[76%, 82%]	0.022
**Monthly Income (tertiles)**										
Low (USD < 387)	654	50%	[46%, 54%]	Reference	12%	[10%, 15%]	Reference	77%	[74%, 80%]	Reference
Medium (USD [387, 700])	600	44%	[40%, 48%]	0.103	10%	[8%, 12%]	0.313	78%	[75%, 81%]	1
High (USD > 700)	727	38%	[35%, 42%]	< 0.001	8%	[6%, 10%]	0.049	78%	[75%, 81%]	1
**Residential Type**										
Rural	825	46%	[42%, 49%]	Reference	9%	[8%, 12%]	Reference	79%	[77%, 82%]	Reference
Urban	1172	42%	[40%, 45%]	0.174	10%	[8%, 12%]	0.878	77%	[74%, 79%]	0.400
**Smoking habit**										
Never	1620	45%	[42%, 47%]	Reference	10%	[9%, 12%]	Reference	70%	[62%, 78%]	Reference
Yes	115	42%	[33%, 51%]	0.604	5%	[2%, 11%]	0.898	76%	[71%, 81%]	0.143
Former	262	39%	[34%, 45%]	0.163	10%	[7%, 14%]	0.248	79%	[77%, 81%]	0.708
**Drinking alcohol habit**										
Never	1416	44%	[42%, 47%]	Reference	10%	[8%, 12%]	Reference	76%	[70%, 80%]	Reference
Yes	280	40%	[34%, 46%]	0.253	9%	[6%, 12%]	0.724	78%	[73%, 83%]	0.708
Former	300	44%	[39%, 50%]	1	10%	[7%, 14%]	0.899	78%	[76%, 80%]	1
**BMI**										
< 20	0	0%	-	-	0%	-	-			
[20, 25]	428	15%	[12%, 19%]	Reference	6%	[4%, 9%]	Reference			
[25, 30]	894	42%	[38%, 45%]	< 0.001	9%	[7%, 11%]	0.248	45%	[43%, 47%]	-
⩾ 30	668	65%	[62%, 69%]	< 0.001	13%	[11%, 16%]	< 0.003	33%	[31%, 33%]	-

Confidence intervals were computed using a binomial score-test. Confidence interval: CI. p-values show differences between groups.

Approximately 59% of the volunteers lived in the urban area. Most participants reported that they had never smoked or consumed alcohol. Approximately 78.2% had excess weight, 44.8% had overweight and 33.5% presented obesity, while only 21.4% had normal BMI. Additionally, 68.8% had abdominal obesity.

The prevalence of MetS was 42% for male and 44% for female participants. Metabolic syndrome had a higher prevalence in subjects older than 50 years of age with none- or primary level of education and low economic income, Table 1. There were not significant differences in MetS prevalence in subjects residing in urban vs rural areas, or smoking and drinking habits, Table 1. Also, MetS was significantly greater in subjects with obesity, followed by subjects with overweight compared to normal weight individuals, Table 1. As expected, individuals with MetS more frequently had dysglycemia and dyslipidemia than individuals without the syndrome, Table 2.

**Table 2:** Clinical characteristics of individuals with or without metabolic syndrome, type-2 diabetes, and excess weight.

Variable	Presence of Metabolic Syndrome	Absence of Metabolic Syndrome	p-value	Presence of Type-2 Diabetes	Absence of Type-2 Diabetes	p-value	Presence of Overweight/Obesity	Absence of Overweight/Obesity	p-value
**SBP (mmHg)**	130 (18)	116 (15)	< 0.001	127 (20)	122 (18)	0.003	124 (18)	116 (18)	< 0.001
**DBP (mmHg)**	83 (11)	76 (9)	< 0.001	81 (10)	79 (11)	0.016	80 (10)	75 (10)	< 0.001
**Glucose (mg/dL)**	113 (38)	94 (20)	< 0.001	166 (64)	95 (11)	< 0.001	104 (32)	96 (27)	< 0.001
**Triglycerides (mg/dL)**	237 (128)	153 (85)	< 0.001	219 (136)	187 (111)	0.004	198 (116)	160 (101)	< 0.001
**LDL (mg/dL)**	117 (38)	114 (34)	0.109	122 (42)	115 (36)	0.032	117 (36)	110 (36)	0.002
**HDL (mg/dL)**									
Male	53 (43)	55 (13)	0.372	54 (12)	54 (31)	0.860	54 (33)	57 (14)	0.136
Female	54 (15)	64 (16)	< 0.001	60 (19)	59 (16)	0.622	58 (15)	65 (20)	< 0.001
**WHR**									
Male	0.97 (0.06)	0.92 (0.06)	< 0.001	0.97 (0.07)	0.94 (0.06)	0.004	0.96 (0.06)	0.91 (0.06)	< 0.001
Female	0.9 (0.06)	0.86 (0.07)	< 0.001	0.9 (0.07)	0.88 (0.07)	< 0.001	0.89 (0.07)	0.84 (0.06)	< 0.001

Data are the mean ± standard deviation (SD).

Systolic blood pressure: SBP; Diastolic blood pressure: DBP; Waist to hip ratio: WHR.

p values show statistical differences between subjects with or without the conditions.

The prevalence of T2D was 9% for male and 10% for female participants; T2D was significantly more prevalent in subjects older than 50 years of age and low economic income, Table 1. Prevalence of T2D was not significantly associated with residency, or smoking and alcohol consumption, Table 1. T2D was two times more frequent in subjects with obesity than in individuals with normal BMI. Like the observations with MetS, individuals with T2D more frequently presented dyslipidemia than individuals without diabetes, Table 2. In addition, the prevalence of overweight and obesity was significantly higher in women, older than 50 years of age, with secondary or higher education, Table 1. Overweight and obesity were significantly more common in individuals with higher levels of education than in subjects with non- or primary levels. As shown in Table 2, subjects with overweight/obesity had statistically significant higher serum concentrations of glucose and lipid metabolites than subjects with normal weight.

### Association of animal and plant protein intake with cardio-metabolic conditions

**Fig 1:**
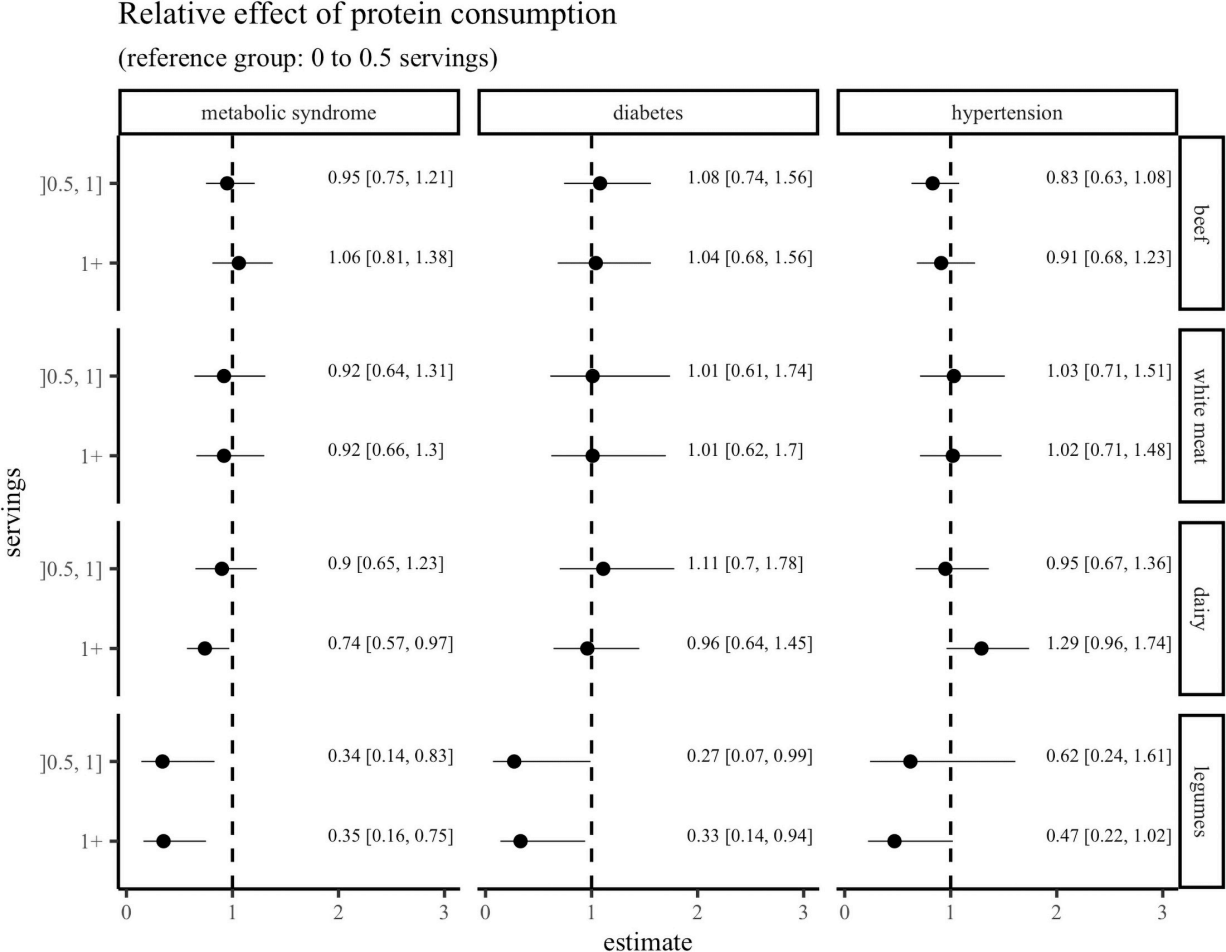
Association between consumption of protein- rich foods and metabolic syndrome, type-2 diabetes, and hypertension. Odds ratios with their 95% confidence intervals for each clinical condition are plotted for type and level of servings of foods. The logistic regression models used are adjusted for: sex, age, body mass index, waist to hip ratio, urban/rural residency, education, income, smoking and drinking habits, energy from diet (including carbonated beverages), physical activity. To analyze protein intake the following categories were used: low, ≤ 0.5; moderate 0.51 to 1, and high >1 serving per day; standard serving sizes (such as a glass of milk, a cup of yoghurt, or a slice of cheese) were assigned for dairy and 150 g for cooked legumes. The reference group considered for comparisons was the consumption of ≤ 0.5 servings.

To assess the effect of the consumption of protein rich foods on cardio-metabolic conditions, we estimated protein intake coefficients and the presence of MetS, T2D, and hypertension. Fig 1 shows that beef and white meat consumption were not associated with the prevalence of MetS, T2D or hypertension. However, the intake of more than one portion of dairy per day showed a protective effect for the presence of MetS, but not, for the presence of T2D or hypertension. In addition, there was a statistically significant inverse association between legume consumption and the presence of MetS and T2D; legume intakes greater that 0.5 servings per day had lower odds in the probability to present MetS and T2D, Fig 1. Consumption of more than 1 portion of legumes showed a tendency to improve the odds in the probability to present hypertension, 0.47, 95% CI [0.22, 1.04], Fig 1.

A closer analysis of a potential relationship between legume consumption and cardiometabolic risk factors did not show significant trends. However, we observed that increasing consumption of legumes was associated with lower systolic and diastolic blood pressure, Table 3. Moreover, we observed similar consumption of macronutrients, (carbohydrates, fats, and proteins) in subjects with or without MetS, T2D, and hypertension (See Online resource: Macronutrients).

**Table 3:** Legume intake and metabolic syndrome clinical characteristics.

Variable	Low ≤ 0.5	Moderate 0.51–1	High > 1	p-value
**SBP (mmHg)**	125 (2.7)	124 (1.9)	122 (0.8)	0.448
**DBP (mmHg)**	82 (1.7)	82 (1.2)	80 (0.5)	0.384
**WHR**	0.9 (0.010)	0.89 (0.007)	0.91 (0.003)	0.033
**BMI (Kg/m**^**2**^**)**	28 (0.7)	29 (0.5)	28 (0.2)	0.384
**HDL (mg/dL)**	57 (3.6)	56 (2.5)	55 (1)	0.887
**LDL (mg/dL)**	107 (6.2)	117 (4.4)	117 (1.8)	0.448
**Triglycerides (mg/dL)**	219 (19.4)	186 (13.8)	198 (5.6)	0.448
**Glucose (mg/dL)**	101 (5.2)	100 (3.7)	102 (1.5)	0.887

Data indicate adjusted means ± standard deviation (SD) of clinical characteristics in function of legume intake, low, moderate, and high, after adjusting for: sex, age, BMI, WHR, urban/rural residency, education, income, smoking and drinking habits, energy from diet, physical activity, and intake of food rich in protein (beef, dairy, and white meat).

Systolic blood pressure: SBP; Diastolic blood pressure: DBP; Waist to hip ratio: WHR; Body mass index: BMI. p-values indicate significant linear trends between legume consumption and clinical variables.

## Discussion

Here we demonstrated that overweigh and obesity and their associated metabolic conditions MetS and T2D are highly prevalent in urban and rural communities in the Province of Pichincha, Ecuador. Excess weight, MetS, and T2D were more common in women older than 50 years of age. MetS and T2D were more common among individuals with none- or primary level of education, low economic income, and with obesity. Metabolic syndrome was more frequent in the rural areas whereas T2D in the urban areas. Of notice, overweight and obesity were more frequent in individuals with higher levels of education. We also observed an inverse association between the consumption of dairy and legumes, rich in proteins, and the prevalence of MetS and T2D. These data should be considered to identify at risk populations and to implement policies to promote protein (animal and plant) consumption.

Cardiometabolic diseases, obesity, MetS, and T2D, are the main causes of morbidity and mortality worldwide. The metabolically related alterations present in MetS are risk factors for the presence of T2D [18]. Metabolic syndrome in Latin America is an increasing health problem, the CARMELA study carried out between 2003 and 2005 established a prevalence that ranged from 14% in Quito to 27% in Mexico City [19]. Another study carried out between 2003 and 2006 in several Central American countries established a MetS-prevalence between 23% in Honduras and 35.1% in Costa Rica [20]. According to the Ecuadorian ENSANUT survey in 2012, the prevalence of MetS was 27% among 10–59 years of age individuals [5]. It is possible that the prevalence of MetS could have increased since several of its components, such as obesity, hypertension, dysglycemia, and dyslipidemia have increased in the last 8 years [21]. Results in the present study showed an estimated prevalence of MetS of 42% which is greater than the ones previously reported in Ecuador, although our estimations could be attributed to the grater age of the studied populations [5, 21]. Here subjects between 35 and 70 years old were included while the ENSANUT included 10–59 years of age individuals. However, the differences in the prevalence of MetS observed in the different studies discussed here can also be related with the methods and diagnostic criteria used, and the sociodemographic characteristics of the populations, and geographic residency which can determine changes in lifestyles, access to medical services, education, work sources, food availability, and urbanization [17].

In Ecuador, there has been an increasing prevalence of T2D during the last few years [21]; the ENSANUT study reported a prevalence of 1.7% in a population aged 10–59 years [5] and a more recent survey in 2018 reported a prevalence of 7.8% in subjects 18 to 69 years of age [21]. Considering the residency of the study population, the prevalence of T2D was higher in the urban area according to the ENSANUT study. Although the present study is limited to the province of Pichincha, a 10% prevalence was observed, being slightly greater in the urban area. These data also indicate that the prevalence of T2D is increasing in Ecuador. Together, these studies indicate that metabolic diseases are increasing in Ecuador and that it is necessary to implement novel intervention strategies to ameliorate the negative impact in the health and economics of the country.

Loss in skeletal muscle mass and function has been associated with increased morbidity and mortality [22, 23]. The association of sarcopenia with metabolic syndrome has been reported [24]. The intake of dietary protein has been associated with muscle mass and other body composition health benefits [25]. Thus, diets rich in animal and plant proteins such as dairy and legumes are associated with decreased morbidity and mortality, decreasing the risk for sarcopenia. In a recent report that included a systematic review and a dose-response meta-analysis of cohort studies with 715,128 participants for a follow-up period of 3.5 to 32 years, total protein intake was associated with a lower risk for all-cause mortality [26]. Furthermore, consumption of plant protein was associated with a lower risk of all-cause mortality and CVD mortality [26]. Similarly, a large prospective study has shown that high plant protein consumption is associated with lower risk of overall and CVD mortality [27]. In the present study, we observed an inverse association between consumption of legumes and the prevalence of MetS and T2D; also, the presence of MetS was inversely associated with dairy intake. These results are consistent with data from the international PURE cohort study that showed a lower risk of non-cardiovascular and total mortality in individuals with high fruit, vegetable, and legume intake [10]. Specifically, legume intake was inversely associated with non-cardiovascular death and total mortality [10]. Also, previous data from the international cohort of the PURE study that assessed the association between total dairy intake with mortality and cardiovascular disease, during a follow-up of period of 9.1 years, showed that consumption of more than 2 servings of total dairy per day was associated with a lower risk of total mortality, non-cardiovascular mortality, cardiovascular mortality, major cardiovascular disease, and stroke compared with no dairy intake [13]. Together, these studies indicate that consumption of foods rich in proteins could potentially decrease the risk to develop cardio-metabolic diseases and ameliorating the cumulative burden of mortality attributable to these conditions. Dietary proteins could maintain and improve muscle mass and function decreasing the risk of sarcopenia and consequently improve internal metabolism. The beneficial effect observed with high protein consumption could be explained by the effect of skeletal muscle maintenance and decreased fat accumulation to reduce the risk of cardiometabolic diseases.

Several clinical *and in vitro* studies could explain how proteins could positively affect human health. Consumption of plant derived proteins have been associated with decreased blood pressure, decreased obesity and improvement of lipid profile [28, 29]. Here, we observed a non-significant trend of lower blood pressure with high legume intake. Previously, we have shown that consumption of *Lupinus mutabilis* Sweet, a commonly consumed Andean legume rich in proteins, improves insulin receptor sensitivity and lowers blood glucose levels in patients with dysglycemia and T2D [30, 31]. The mechanism of action of hydrolyzed proteins extracted from *L*. *mutabilis* include inhibition of of dipeptidyl peptidase-IV (100%) activity, increase translocation of glucose transporter type-4 and reduction of gluconeogenesis in vitro [12]. Studies with hydrolysates from protein isolates from another legume like *Phaseolus vulgaris* L have also generated peptides that can modify glucose metabolism [32]. Similarly, studies with hydrolysates obtained from milk casein and whey proteins stimulate the expression of proglucagon, pro-glucagon like peptide 1 (GLP-1), and cholecystokninin; and the release of GLP-1 in the entero-endocrine STC (pGIP/Neo) cell line [33]. These hormones are related with the regulation of internal metabolism. It will be important to characterize local Andean plant and animal protein derived hydrolysates and study their potential biological activities. New strategies based on the use of local produce to prevent and treat metabolic conditions could improve the management of chronic diseases in the general population.

We acknowledge some limitations of the present study. Macronutrient intake was calculated through FFQ which limits specific analysis regarding the quality of macronutrient content. For example, we did not observe differences in the amount of carbohydrates, fats, and proteins in individuals with or without, MetS, T2D, and overweight/obesity. The quality of the diet, rather than its quantity, influences the gut microbiota composition, which has been associated with metabolic conditions such as MetS, T2D, and obesity [34]. On the other hand, the strengths of this study include the large number of participants; the study was carried out in the second largest province of the country; and it assessed local food consumption and the risk of cardio-metabolic diseases. Also, this study follows the methodology of the international PURE cohort, hence these results can be compared worldwide.

## Conclusions

Together present results indicate that MetS and T2D are highly prevalent in urban and rural communities and that consumption of plant and animal foods, rich in proteins, could decrease the frequency of these common public health pathologies. It is important to recommend the consumption of protein rich foods like dairy and original Andean legumes which are part of traditional diets, for the general population.

## Supporting information

S1 File (DOCX)
