# Supplementary material for: Prevalence of metabolic syndrome and diabetes mellitus type-2 and their association with intake of dairy and legume in Andean communities of Ecuador
Source: PLoS One. 2021 Jul 23;16(7):e0254812. doi: 10.1371/journal.pone.0254812 (PMC8301611; doi:10.1371/journal.pone.0254812)
Supplement: S1 File — (DOCX) [file pone.0254812.s001.docx]

S1 File with supplementary files at:

***Supplementary files are uploaded in the following address:***

https://figshare.com/projects/Metabolic_Syndrome_Tables_and_Supporting_Information_v2_0/116943
